# Unraveling the mechanisms of high-level gait control in functional gait disorders

**DOI:** 10.1007/s00702-024-02829-4

**Published:** 2024-09-06

**Authors:** Angela Sandri, Chiara Bonetto, Mirta Fiorio, Francesca Salaorni, Giulia Bonardi, Christian Geroin, Nicola Smania, Michele Tinazzi, Marialuisa Gandolfi

**Affiliations:** 1 Department of Neurosciences, Biomedicine and Movement Sciences, University of Verona, P. le L.A. Scuro, 10, Verona, 37134 Italy; 2 Department of Surgery, Dentistry, Pediatrics and Gynecology, University of Verona, Verona, Italy; 3 Neuromotor and Cognitive Rehabilitation Research Centre (CRRNC), University of Verona, Verona, Italy

**Keywords:** Cognition, Gait, Locomotion, Attention, Functional motor disorders, Risk of falls

## Abstract

Functional gait disorders (FGDs) are a disabling subset of Functional Neurological Disorders in which presenting symptoms arise from altered high-level motor control. The dual-task paradigm can be used to investigate mechanisms of high-level gait control. The study aimed to determine the objective measures of gait that best discriminate between individuals with FGDs and healthy controls and the relationship with disease severity and duration. High-level spatiotemporal gait outcomes were analyzed in 87 patients with FGDs (79.3% women, average age 41.9±14.7 years) and 48 healthy controls (60.4% women, average age 41.9±15.7 years) on single and motor, cognitive, and visual-fixation dual tasks. The area under the curve (AUC) from the receiver operator characteristic plot and the dual-task effect (DTE) were calculated for each measure. Dual-task interference on the top single-task gait characteristics was determined by two-way repeated measures ANOVA. Stride time variability and its standard deviation (SD) failed to discriminate between the two groups in single and dual-task conditions (AUC<0.80 for all). Significant group x task interactions were observed for swing time SD and stride time on the cognitive dual tasks (p<0.035 for all). Longer disease duration was associated with poor gait performance and unsteadiness in motor and cognitive DTE (p<0.003) but improvement in stride length and swing time on the visual dual tasks (p<0.041). Our preliminary findings shed light on measures of gait automaticity as a diagnostic and prognostic gait biomarker and underline the importance of early diagnosis and management in individuals with FGDs.

**Supplementary Information:**

The online version contains supplementary material available at 10.1007/s00702-024-02829-4.

## Introduction

Functional gait disorders (FGDs) are among the most debilitating symptoms in 23–45% of patients with functional motor disorders (FMD) (Baizabal-Carvallo et al. 2020; Tinazzi et al. 2021d). Despite science’s progress, FMD remains misunderstood and inadequately treated, increasing the disease burden for patients and caregivers (Tinazzi et al. 2021a; Watson et al. 2023). Diagnosis relies on clinical assessment to identify symptom inconsistency, where gait patterns change with interference, and incongruence, where patterns do not match those seen in neurological disorders like stroke or Parkinson’s disease and Multiple Sclerosis (Nonnekes et al. 2020). Understanding FGDs pathophysiology and refining clinical assessment are unmet needs to improve patient management and reduce long-term disability (Gandolfi et al. 2023a).

The pathophysiology of FGDs shares common features with functional neurological disorders (FND), including altered attentional focus, ingrained misconstrued beliefs and expectations, and a disrupted sense of agency (Marotta et al. 2017; Tinazzi et al. 2021c; Hallett et al. 2022). This condition is thought to involve heightened limbic system activity and encapsulate an internal model of symptoms within a predictive coding framework from abnormal brain network functioning rather than from structural brain damage (Hallett et al. 2022). FGDs patterns, such as slow walking, astasia-abasia, and knee buckling, often change when the individual is distracted or engages in non-physiological movements, suggesting compromised higher-level gait control (Fung 2016; Nonnekes et al. 2020; Tinazzi et al. 2020; Gandolfi et al. 2023a). Indeed, gait is an intricate activity integrating lower and higher-level functions, including cognitive, visuospatial, somatosensory, and motor planning abilities. It involves the central and peripheral nervous systems to regulate muscle activation, rhythm, and movement patterns, fine-tuned by feedback from the visual, vestibular, and proprioceptive systems. Walking often demands significant cognitive resources, especially when adjusting speed, direction, or navigating challenging environments, requiring executive functions and focused attention (Yogev-Seligmann et al. 2008; Al-Yahya et al. 2011; Takakusaki 2013; Mirelman et al. 2018).

The dual-task paradigm is useful for exploring the relationship between gait and cognition (Al-Yahya et al. 2011; Amboni et al. 2013; Plummer et al. 2015). Dual-tasking can impair performance, reduce automaticity, and increase fall risk in healthy individuals, the elderly, and those with neurological conditions like multiple sclerosis, stroke, Parkinson’s disease, and dementia (Al-Yahya et al. 2011; Amboni et al. 2013; Raffegeau et al. 2019; Chen et al. 2020). Preliminary evidence suggests that the dual-task paradigm could also help understand postural and gait disturbances in FMD with FGDs (Gandolfi et al. 2021, 2023a). In our preliminary exploration of dual-tasking’s impact on gait in a small cohort of FMD with FGDs versus healthy controls (HC), we found poorer gait performance, automaticity, and steadiness in patients during single-task walking (Gandolfi et al. 2023a). While dual-tasking (cognitive, motor, and visual) further impacted gait performance, it did not significantly affect automaticity and steadiness, differing from patterns seen in neurological diseases and highlighting the role of higher-level gait control in FMD with FGDs (Gandolfi et al. 2023a). Building on this foundation, we expanded our previous study to a larger sample with a robust statistical methodology (Vitorio et al. 2021; Gandolfi et al. 2023a). Although FGDs are primarily diagnosed through clinical pattern recognition, expanding gait analysis and refining dual-tasking methods are necessary. Stride time variability shows promise as a diagnostic biomarker, underscoring the need for further research into spatial-temporal gait parameters and specific biomarkers for FMD with FGDs.

## Methods

### Study design

For this cross-sectional observational study, a convenience sample of 87 people with FMD (Gupta and Lang 2009) and FGDs (age 41.9 ± 14.7 years; 80.5% women) and 48 HC (age 41.9 ± 15.7 years; 60.4% women) was enrolled from the Parkinson’s Disease and Movement Disorders Unit of the AOUI of Verona, Italy.

### Participants

Inclusion criteria were: age 18 years or older, a clinically definite diagnosis of FMD (Gupta and Lang 2009), lower limb functional motor symptoms (at least one of the following: tremor, weakness, jerks, dystonia) and sensory symptoms (Tinazzi et al. 2021d), presence of FGDs (assessed by an expert neurologist) including slow gait, astasia-abasia, knee buckling, paraparetic gait, ice walking gait, hemiparetic gait, tightrope gait and others (Tinazzi et al. 2021d); Mini-Mental State Examination (MMSE) score ≥ 24/30 (Folstein et al. 1975). Exclusion criteria were persistent dissociative seizures, need for an assistive device to maintain upright posture, other comorbidities that could interfere with gait performance, use of neuroleptics, and a physical impairment precluding signing the informed consent form for participation in the study. Duration and severity of FMD symptoms were quantified using the objective-rated Simplified Functional Movement Disorders Rating Scale (S-FMDRS, range, 0–54; higher scores indicate worse rating) (Nielsen et al. 2017). All patients were assessed before undergoing a targeted multidisciplinary rehabilitation program.

### Compliance with ethical standards

All participants gave their written, informed consent to participate. The study was carried out following the tenets of the Helsinki Declaration and approved by the local Ethics Committee (Prog. 3571CESC - JP-VR-19).

### Gait assessment

Spatial-temporal gait parameters were collected at a self-selected comfortable speed on a 7.92-m electronic walkway (GAITRite, USA; Gandolfi et al. 2023a). Nine spatial-temporal gait measures (average, standard deviation and/or variability) spanning lower and high-level gait control and sensitive to the dual-task effect were extracted (Al-Yahya et al. 2011; Vitorio et al. 2021; Gandolfi et al. 2023a) as follows: Gait speed (cm/s), Stride length (cm), Stride time (s), Stride time variability (%), Swing time (s) and %, Swing time variability (%), Double support (s), and Step duration (s). The complete list and definition of objective measures are presented in Supplementary Table 1.

### Experimental protocol

**Fig. 1:**
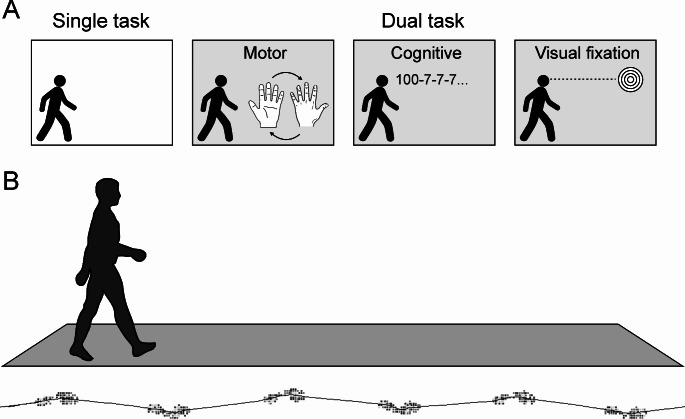
Experimental Set-up **A**) The participants were assessed while engaged in a single task (ST), a motor dual-task (mDT), a cognitive dual-task (cDT), and a visual-fixation task (vDT), without the aid of a walking device. During the ST, the participants walked at a self-selected comfortable speed. The mDT involved walking while performing pronation-supination movements with the right hand on the right thigh at a frequency of 1 Hz, without auditory cues. During the cDT, the participants performed serial subtraction by sevens starting from 100. On the vDT, they walked while maintaining their gaze on a “destination-focused” fixation circle positioned at eye level ahead of them. The participants were instructed to prioritize the motor, cognitive, or visual fixation task before each task. The tasks were executed in the sequence described. **B**) Spatial-temporal gait parameters were assessed while the participants walked along a 7.92-m electronic walkway. Each trial was conducted three times to average gait parameters and reduce potential bias

The experimental setup is illustrated in Fig. 1. As done previously, four conditions were tested: single task (ST), motor (mDT), cognitive (cDT), and a visual-fixation dual-task (vDT) (Gandolfi et al. 2023a). In the single-task condition, participants were instructed to walk at their self-selected comfortable speed without performing any adjunctive task (Gandolfi et al. 2023a). The mDT and the cDT entailed walking down the walkway while executing prono-supination movements with the right hand and serially subtracting seven starting from 100, respectively (Gandolfi et al. 2023a). The vDT focused on a “destination-focused” fixation circle placed at eye level in front of the participant (Gandolfi et al. 2023a). The ST condition was completed before the dual task-conditions (Vitorio et al. 2021). The dual-task conditions were performed in the same order as described above (Gandolfi et al. 2023a).

### Statistical analysis

Descriptive statistics included the frequency (no, %) for categorical variables and the mean (with standard deviations, SDs) for continuous measures. Comparison between the two groups (patients and HC) was performed using Fisher’s exact test for sex and the t-test for age.

The dual-task effect (DTE) expressed in percentage (%) was computed for each spatial-temporal gait parameter to quantify the interference between the ST and each DT condition (mDT, cDT, vDT) using the following equation (Plummer et al. 2015)

$$\begin{array}{l}\:DTE\:\left(\%\right)\\=\:\frac{Dual\:task\:performace-Single\:task\:performance}{Single\:task\:performance}\:\\\times\:100\end{array}$$

For gait outcomes where higher values indicate better performance (gait speed, swing time, stride length) higher or positive DTE values indicate better performance on the dual-task (DT) compared with the single-task (ST). Conversely, for outcomes where higher values are indicative of worse performance (double support time, swing time variability, stride time variability, step duration, stride time), higher or positive DTE values indicate worse performance on the DT compared with the ST (Plummer-D’Amato et al. 2012; Gandolfi et al. 2023a). Details are given in Supplementary Table 1.

To investigate which spatial-temporal gait measures on ST, DT, and DTE best discriminate between performance for people with FGDs and performance for HC, ROC curve analysis (Robin et al. 2011) was performed: the area under the curve (AUC) values were ordered from highest to lowest [AUC = 0.9–1.0 (excellent); 0.8–0.9 (good); 0.7–0.8 (fair); 0.6–0.7 (poor); 0.5–0.6 (fail)]. The ROC analysis was re-run by stratifying for sex. Finally, sensitivity analysis was performed by deleting the outliers from the sample and re-estimating the AUC values. Repeated measures ANOVA with the factors group (FGD, HC), task (ST, DT), and the interaction group x task was performed for all gait measures, both separately for each pair (ST, mDT), (ST, cDT) and (ST, vDT) and taking the four tasks together (ST, mDT, cDT, vDT).

Correlations between each gait measure and symptom severity (based on the s-FMDRS total score) and disease duration (in years) were explored using Pearson’s coefficient. All tests were bilateral at *p* < 0.05. Statistical analysis was performed using Stata 17 for Windows (Stata Corp, USA).

## Results

### Study sample characteristics

There was a higher proportion of women in the FDG group than in the HC group (80.5% vs. 60.4%; *p* = 0.015), while the average age was about 42 years. The disease duration was less than 2 years in half of the FDG group (Table 1).

**Table 1:** Demographics and clinical characteristics of FGDs patients and healthy controls

Characteristic	FGDs patients *N* = 87	Healthy controls *N* = 48	*p*-value
Women, no. (%) ^§^	70 (80.5)	29 (60.4)	0.015
Age (years), mean (SD)^#^	41.9 (14.7)	41.9 (15.7)	0.997
Disease duration (years), mean (SD) - median	3.4 (4.3) – 2.0	-	-
s-FMDRS total score, mean (SD)°	19.3 (10.3)	-	-

Legend: FGDs, functional gait disorders; no., number; SD, standard deviation; s-FMDRS, simplified Functional Movement Disorders Rating Scale (total score range, 0–54; higher scores indicate worse rating); ^§^ Fisher’s test; ^#^ t -Test; ° 2 missing data

### Single-task and dual-task gait measures

**Fig. 2:**
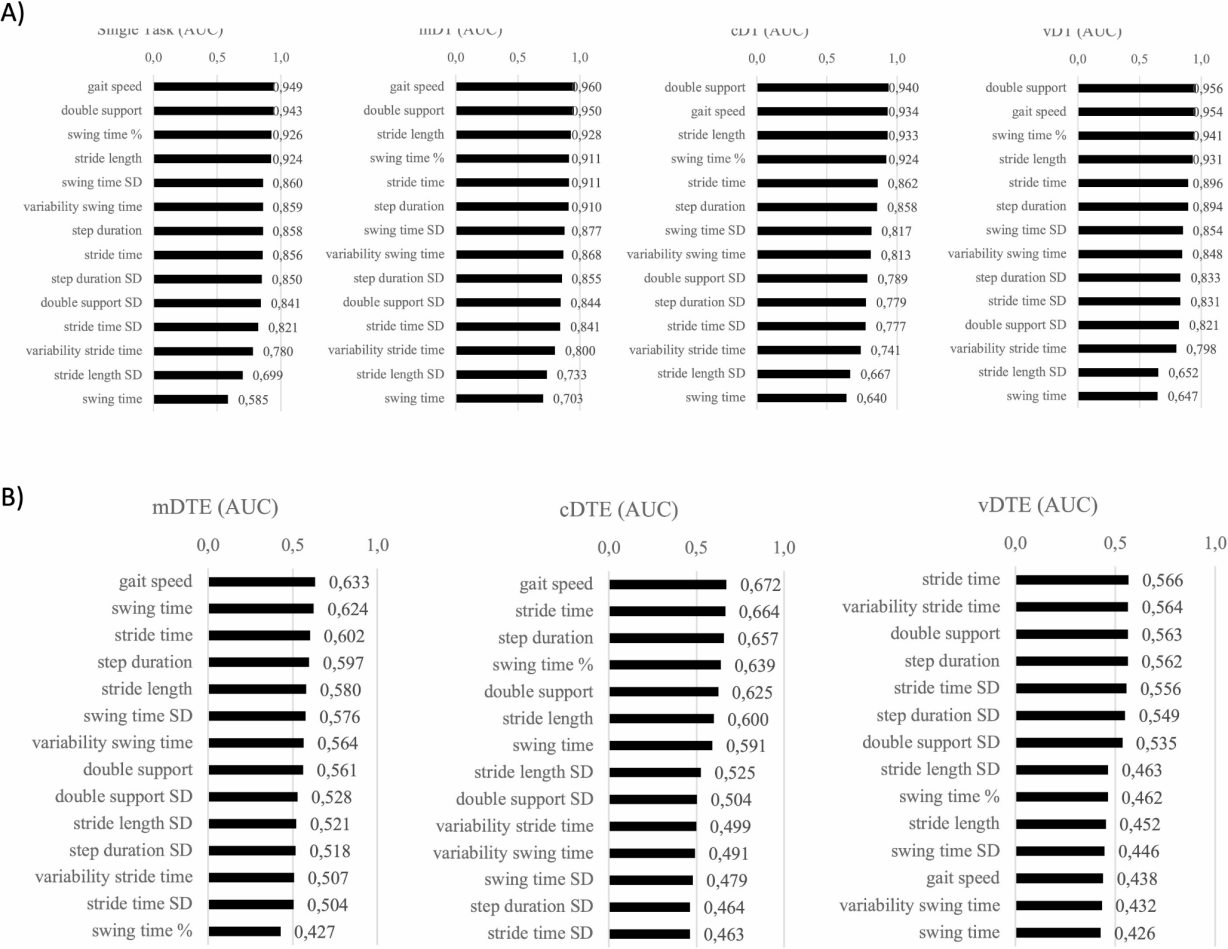
Area under the curve in descending order discriminating between FGD patients and healthy controls. **A**) Area under the curve in descending order for each gait measure discriminating between FGD patients and healthy controls. Legend: m, motor; c, cognitive; v, visual; DT, Dual Task; AUC, Area Under the Curve; SD, Standard Deviation; %, percentage. A rough guide for assessing the utility of a biomarker based on its AUC is: 0.9–1.0 (excellent);0.8–0.9 (good); 0.7–0.8 (fair); 0.6–0.7 (poor); 0.5–0.6 (fail). **B)** Area under the curve in descending order for each dual-task effect (%) gait measure discriminating between FGD patients and healthy controls. Legend: m, motor; c, cognitive; v, visual; DTE, dual-task effect; AUC, area under the curve; SD, standard deviation; %, percentage. A rough guide for assessing the utility of a biomarker based on its AUC is: 0.9–1.0 (excellent); 0.8–0.9 (good); 0.7–0.8 (fair); 0.6–0.7 (poor); 0.5–0.6 (fail)

AUC values were < 0.80 on all tasks for stride time variability, stride length SD, and swing time (s), indicating that they did not discriminate between the two groups for performance measures. Four gait measures discriminated performance between the two groups on the single task at an excellent AUC level (> 0.90): gait speed (AUC 0.949), double support (AUC 0.943), swing time (%) (AUC 0.926), and stride length (AUC 0.924). The same four gait measures similarly discriminated performance between the two groups in dual-task conditions (mDT, cDT, vDT; AUC > 0.90) (Fig. 2). These findings were confirmed when stratified by for sex, with women showing overall higher AUCs (online Supplementary Table 2). The DTE AUCs for the mDT and the cDT for the other gait measures were < 0.70, with the v-DTEs < 0.60 (Fig. 2). Sensitivity analysis was performed by deleting outliers from the sample (11 FGDs and 7 HC) and re-running ROC analysis. The AUC values and their rankings were confirmed (data available from the Authors).

### Dual-task performance

Table 2 presents the two groups’ raw mean and SDs for gait measures. In the comparison between ST and each DT (mDT, cDT, vDT, respectively), only swing time SD and stride time for cDT showed a significant Group $\:\times\:\:$Task interaction (swing time SD cDT: FGDs 0.12, SD 0.12, vs. HC 0.03, SD 0.021, *p* = 0.027; stride time cDT: FGDs group 1.81, SD 0.72, vs. HC 1.21, SD 0.19, *p* = 0.035; Tables 2 and 3), indicating that within those two measures the FGDs group performed worse than the HC only on the cDT. The group effect was significant for all measures, except on the cDT in stride time SD (FGDs 0.40, SD 1.39 vs. HC 0.12, SD 0.28, *p* = 0.067) and stride time variability (FGDs 15.41, SD 30.04 vs. HC 8.76, SD 16.11, *p* = 0.055), meaning that performance on the cDT was similar for both groups (Table 3).

**Table 2:** Raw mean and SDs for gait measures (group, FGDs patients vs. healthy controls; task: single ST vs. dual DT)

Gait measure	Group	ST		mDT		cDT		v DT	
		Mean	SD	Mean	SD	Mean	SD	Mean	SD
**Pace domain**									
Gait speed (cm/s)	FGDs HC	66.68 115.92	28.51 13.58	59.42 110.88	25.81 14.17	52.53 100.43	24.66 20.03	68.09 119.61	27.49 17.04
Stride length (cm)	FGDs HC	89.25 126.99	24.57 11.47	83.49 123.04	24.57 11.94	81.97 119.65	23.56 12.84	90.84 128.31	23.83 12.60
Step duration (s)	FGDs HC	0.77 0.55	0.31 0.05	0.77 0.56	0.22 0.04	0.90 0.61	0.35 0.12	0.77 0.54	0.34 0.05
**Rhythm domain**									
Stride time (s)	FGDs HC	1.51 1.10	0.57 0.09	1.54 1.11	0.44 0.09	1.81 1.21	0.72 0.19	1.50 1.08	0.59 0.10
Swing time (s)	FGDs HC	0.44 0.42	0.08 0.03	0.46 0.42	0.07 0.03	0.49 0.45	0.10 0.05	0.44 0.41	0.06 0.03
Double support (s)	FGDs HC	0.64 0.25	0.55 0.04	0.64 0.27	0.43 0.04	0.81 0.31	0.62 0.06	0.64 0.25	0.59 0.05
**Phase domain**									
Swing time %	FGDs HC	31.49 38.47	6.88 1.01	31.36 37.95	6.08 1.26	29.61 37.31	7.04 1.96	31.37 38.40	6.78 1.30
**Variability measure**									
Swing time SD	FGDs HC	0.08 0.02	0.09 0.01	0.10 0.02	0.15 0.02	0.12 0.03	0.12 0.02	0.08 0.02	0.08 0.02
Swing time variability	FGDs HC	17.05 5.09	17.67 2.78	21.65 5.27	26.79 3.91	23.09 7.22	24.90 4.62	18.21 5.08	17.80 4.01
Step duration SD	FGDs HC	0.17 0.03	0.39 0.02	0.14 0.10	0.16 0.03	0.26 0.09	0.69 0.26	0.24 0.03	0.57 0.02
Double support SD	FGDs HC	0.23 0.03	0.58 0.02	0.16 0.04	0.19 0.06	0.29 0.06	0.75 0.06	0.26 0.03	0.62 0.03
Stride time SD	FGDs HC	0.21 0.06	0.33 0.10	0.19 0.06	0.26 0.11	0.40 0.12	1.39 0.28	0.26 0.05	0.53 0.11
Stride time variability	FGDs HC	10.88 5.21	12.96 9.22	10.76 5.29	12.74 10.43	15.41 8.76	30.04 16.11	13.89 4.84	22.00 9.99
Stride length SD	FGDs HC	5,63 3.74	3.63 1.32	5.12 3.34	3.11 1.09	5.90 4.53	3.28 2.39	5.75 3.64	5.93 1.40

Legend: s, seconds; cm, centimeters; ST, single task; mDT, motor dual task; cDT, cognitive dual task; vDT, visual-fixation dual task; SD, standard deviation; FGDs, functional gait disorders; HC, healthy controls

**Table 3:** ANOVA for gait measures in discriminating performance between FGDs patients and healthy controls (group: FGDs patients vs. healthy controls; task: ST vs. mDT, ST vs. cDT, ST vs. vDT)

		mDT		cDT		vDT	
Gait measure	Main effect / interaction	F	*p*	F	*p*	F	*p*
**Pace domain**							
Gait speed (cm/s)	Group Task Group x Task	157.57 24.13 0.78	< 0.001 < 0.001 0.378	143.84 122.13 0.25	< 0.001 < 0.001 0.618	146.29 3.46 1.05	< 0.001 0.065 0.308
Stride length (cm)	Group Task Group x Task	114.10 21.54 0.75	< 0.001 < 0.001 0.388	108.32 79.39 0.00	< 0.001 < 0.001 0.973	108.22 2.31 0.00	< 0.001 0.131 0.954
Step duration (s)	Group Task Group x Task	39.02 0.13 0.01	< 0.001 0.718 0.929	31.52 27.62 3.37	< 0.001 < 0.001 0.069	30.51 0.01 0.07	< 0.001 0.917 0.799
**Rhythm domain**							
Stride time (s)	Group Task Group x Task	38.70 0.61 0.07	< 0.001 0.437 0.785	34.96 23.63 4.56	< 0.001 < 0.001 **0.035**	29.25 0.24 0.00	< 0.001 0.626 0.974
Swing time (s)	Group Task Group x Task	10.40 2.49 2.43	0.002 0.117 0.122	7.18 39.10 3.45	0.008 < 0.001 0.066	4.98 3.65 0.00	0.027 0.058 0.967
Double support (s)	Group Task Group x Task	33.97 0.02 0.18	< 0.001 0.875 0.675	31.60 12.20 3.26	< 0.001 < 0.001 0.073	26.73 0.02 0.01	< 0.001 0.892 0.913
**Phase domain**							
Swing time %	Group Task Group x Task	60.06 0.82 0.28	< 0.001 0.366 0.598	57.20 22.98 1.29	< 0.001 < 0.001 0.257	55.02 0.14 0.02	< 0.001 0.714 0.892
**Variability measures**							
Swing time SD	Group Task Group x Task	18.67 1.96 1.79	< 0.001 0.164 0.183	21.53 17.52 5.01	< 0.001 < 0.001 **0.027**	25.29 0.00 0.02	< 0.001 0.973 0.899
Swing time variability	Group Task Group x Task	22.22 3.07 2.62	< 0.001 0.082 0.108	22.67 12.17 2.79	< 0.001 0.001 0.097	29.77 0.21 0.22	< 0.001 0.646 0.638
Step duration SD	Group Task Group x Task	12.95 0.54 0.52	< 0.001 0.466 0.471	3.95 5.56 0.16	0.049 0.020 0.692	11.83 0.45 0.53	< 0.001 0.502 0.470
Double support SD	Group Task Group x Task	11.24 0.48 0.94	0.001 0.488 0.334	5.34 3.01 0.49	0.022 0.085 0.487	11.29 0.09 0.06	0.001 0.766 0.815
Stride time SD	Group Task Group x Task	12.19 0.21 0.25	0.001 0.644 0.616	3.40 2.15 0.55	0.067 0.145 0.459	12.89 0.33 0.47	< 0.001 0.568 0.495
Stride time variability	Group Task Group x Task	9.61 0.00 0.01	0.002 0.986 0.926	3.74 5.45 0.08	0.055 0.021 0.778	10.79 0.60 0.98	0.001 0.438 0.323
Stride length SD	Group Task Group x Task	19.73 2.44 0.03	< 0.001 0.120 0.858	12.74 3.27 0.80	< 0.001 0.073 0.373	10.73 0.00 0.06	0.001 0.999 0.803

Legend: s, seconds; cm, centimeters; ST, single task; mDT, motor dual task; cDT, cognitive dual task; vDT, visual-fixation dual task; SD, standard deviation; FGDs, functional gait disorders; HC, healthy controls. In bold only significant Group x Task interaction

### Correlations between gait measures, FMD severity, and disease duration

There was no significant correlation between the s-FMDRS total score and any gait measure (ST, mDT, cDT, vDT, mDTE, cDTE, vDTE), whereas disease duration correlated significantly only with DTEs. In detail, two motor DTEs were positively correlated (swing time variability, r 0.311, *p* = 0.003; swing time SD, r 0.288, *p* = 0.007), together with two cognitive DTEs (swing time variability, r 0.254, *p* = 0.018; double support SD, r 0.215, *p* = 0.046). There was a negative correlation between four visual-fixation DTEs and disease duration (step duration, r -0.220, *p* = 0.041; stride length, r -0.236, *p* = 0.028; stride time, r -0.234, *p* = 0.029; swing time, r -0.288, *p* = 0.007).

## Discussion

This is the first study to compare a broad set of objective measures of spatial-temporal gait parameters to determine which measures on single and dual tasks could best discriminate performance between individuals with FMD and FGDs, and HC. The key findings are threefold.

First, variability in stride time did not effectively differentiate performance between the two groups in either the single-task or the dual-task (DTs) conditions, as evidenced by an area under the curve (AUC) < 0.80. This finding supports our previous results (Gandolfi et al. 2023a) and primary hypothesis. It also suggests that although gait performance, automaticity, and steadiness were poorer for the FGDs group across all tasks (Gandolfi et al. 2023a), the percentage change in gait automaticity (as measured by stride time variability) was comparable between the two groups. Stride time variability could conceivably provide a biomarker for the diagnosis of FGDs as a measure of gait automaticity integrity in elderly patients prone to falling and in patients with neurological disorders (e.g., Parkinson’s disease and multiple sclerosis) since dual-tasking impacts on gait automaticity (higher stride time variability) (Yogev et al. 2005; Springer et al. 2006; Al-Yahya et al. 2011). The next step is to determine whether these effects can be observed in other neurological disorders and if our results may change over time based on factors such as severity, disease duration, and the impact of a targeted rehabilitation protocol, which could be further assessed in future clinical trials.

Second, the group with FMD and FGDs performed worse than the HC on swing time SD and stride time, which was evident only on the cDT but not on the motor or visual DTs. Gait performance on these two dual tasks of the group with FMD and FGDs was like that of the HC, suggesting that the cognitive task exerts a detrimental effect. Furthermore, on the cDT, measures of gait automaticity (stride time variability and stride time SD) were like those observed in the HC, indicating that on the cDT (but not on the motor or the visual DTs), physiological gait automaticity control was similar for both groups. The cDT is a mental tracking task during which subjects must hold information in memory while performing a mental process (Al-Yahya et al. 2011). Indeed, stride time variability under mental tracking dual tasks could serve as a diagnostic and prognostic gait biomarker in FGDs. Evidence from a direct comparison between the cognitive DTs suggests that evaluating gait performance (gait speed and stride length) and automaticity (stride time variability) under mental tracking dual-task conditions may differentiate between HC and subjects with neurological deficits (Al-Yahya et al. 2011). Moreover, performance by people with neurological disorders on mental tracking is known to be decreased on other cognitive dual tasks, such as verbal fluency and working memory (Al-Yahya et al. 2011). Our observations in FGDs suggest that evaluation of gait automaticity (stride time variability) on mental tracking DTs may help identify FMD patients with the structural integrity of automaticity gait control in FGDs (Leitner et al. 2007). We speculate that a mental tracking task may be particularly effective in redirecting attention in FGDs compared to other DTs (mDT and vDT) and might help inform specific rehabilitation interventions (Gandolfi et al. 2023b).

Finally, the impact of DTs may be modulated by disease duration. It is important to note that the median disease duration in our sample is two years, classifying it as a chronic condition. Including patients with longer disease durations in future studies could enhance the generalizability of our findings, especially given that many patients today have extended disease courses. Nevertheless, as the disease progresses, the benefits of motor and cognitive DTs on stability-related measures (e.g., swing time variability, double support time, swing time, swing time SD, stride length) diminish. Concurrently, the visual-fixation DT might enhance only some aspects of gait performance, namely, step duration and stride time. This finding holds implications for managing patients with FGDs and underscores the need for early diagnosis (Tinazzi et al. 2021a). Furthermore, personalized treatment plans and rehabilitation strategies could be adjusted for disease duration. To this end, a study with FMD and FGDs stratified by short and long disease duration could help corroborate such inferences.

This is the first study to extensively explore a comprehensive range of objective spatiotemporal gait parameters in a cohort of patients with FMD and FGDs and an HC group, expanding our previous study to a larger sample with a more robust statistical methodology using receiver operating characteristics (ROC) curve analysis to enhance the accuracy and credibility of our results, and incorporating additional spatial-temporal gait measures under various attentional conditions, both in single and dual-task scenarios, for ROC curve analysis (Al-Yahya et al. 2011; Robin et al. 2011; Vitorio et al. 2021; Gandolfi et al. 2023a).

Due to our research’s novelty and specificity, direct comparisons with previous studies are not feasible. Building upon Vitorio’s findings in individuals with Parkinson’s Disease (Vitorio et al. 2021), we believe that conventional measures such as gait speed and stride length must be revised to capture the FMD-specific functional nature of gait disturbances accurately and that a more focused investigation into specific high-order levels of gait control is warranted for FGDs. Specifically, our study suggests that stride time variability serves as a more indicative biomarker of the structural integrity of gait neurophysiology and, as such, should be considered a key gait biomarker in the context of FGDs. Furthermore, these results may be linked to alterations in specific neural circuits. While it is known that FMD involves sensorimotor, reward, and emotion circuits (Waugh et al. 2023), no studies have focused solely on gait impairments, partly due to the complexity of FMD, which often presents with multiple motor symptoms and the rarity of isolated FGDs (Tinazzi et al. 2021b). Future research should also investigate spatial-temporal measures in the different FGDs patterns to better understand their pathophysiology and develop personalized rehabilitation strategies.

Our study underscores the value of incorporating objective gait measurements into the clinical assessment of patients with FGDs and utilizing simple gait analysis tools for quantitative gait assessment. The key strengths of our study are the large sample of patients with a definitive diagnosis of FMD, the use of an easily implementable device for gait assessment under a validated dual-task protocol, and the statistical methodology supporting the study’s validity.

The study also has limitations: the absence of neuropsychological assessment and the need for more data on turning and movements of the upper limbs, trunk, and gaze. Additionally, the use of benzodiazepines should be documented.

In conclusion, our findings reinforce the notion of stride time variability as a potential prognostic and diagnostic biomarker for gait impairment in FGDs and using motor and visual fixation dual tasks to retrain correct movement patterns. Early diagnosis and management of these patients are also important.

## Electronic supplementary material

Below is the link to the electronic supplementary material.

Supplementary material 1
