# Supplementary material for: Unraveling the mechanisms of high-level gait control in functional gait disorders
Source: J Neural Transm (Vienna). 2024 Sep 6;132(1):95–104. doi: 10.1007/s00702-024-02829-4 (PMC11735589; doi:10.1007/s00702-024-02829-4)
Supplement: Supplementary file 1 — Supplementary material 1 [file 702_2024_2829_MOESM1_ESM.docx]

**Supplementary Information**

**Title: Unraveling the Mechanisms of High-Level Gait Control in Functional Gait Disorders**

**Authors:**

Angela Sandri^1^

Chiara Bonetto^1^

Mirta Fiorio^1^

Francesca Salaorni^1^

Giulia Bonardi^1^

Christian Geroin^2^

Nicola Smania^1,2,3^

Michele Tinazzi^1*^

Marialuisa Gandolfi^1,2,3*^

***** Correspondence information:

Prof. Michele Tinazzi. Department of Neurosciences, Biomedicine and Movement Sciences, University of Verona, Italy. Phone: +39 045 8122601; P. le L.A. Scuro, 10, 37134 Verona, Italy. E-mail: [michele.tinazzi@univr.it](mailto:michele.tinazzi@univr.it)

Prof. Marialuisa Gandolfi. Department of Neurosciences, Biomedicine and Movement Sciences, University of Verona, Italy. Phone: +39 045 8124349; P. le L.A. Scuro, 10, 37134 Verona, Italy. E-mail: [marialuisa.gandolfi@univr.it](mailto:marialuisa.gandolfi@univr.it)

**Supplementary Table 1: Gait Parameters description.**

| **Pace domain** |
| --- |
| **Gait speed (cm/s).**  **Definition-** Unit of distance (cm) per time (s).  **Marker -** General indicator of functional performance, where reduced speed may predict falls^53,54^; associated with co-morbidity, impairments, and cognitive decline in the elderly^55^.  **Neural control -** Gait speed control areas may be interlinked with networks of higher-level cognitive functions like executive function that involve the pre-frontal cortex.  **Modulation during dual task -** Slower on the DT than on the ST in healthy controls and people with neurological disease ^25,40^.  **DTE** - Higher or positive DTE indicates better performance on the DT than on the ST.  **Stride length (cm).**  **Definition -** Linear distance covered during a complete walking cycle - the distance between successive placement of the same foot during walking.  **Marker -** Important metric in assessing walking performance, also in relation to neurological conditions^56,57^.  **Neural control -** Thought to be controlled by the cortico-basal ganglia circuit via the thalamus^25^.  **Modulation during dual task -** Slower on the DT on the ST in healthy controls and people with neurological disease^25,40^.  **DTE -** Higher or positive DTE indicates better performance on the DT than on the ST.  **Step duration (s).**  **Definition -** Length of time it takes to complete one step (from one heel strike to the next heel strike).  **Marker -** Parameter changes in relation to the trunk’s ability to adapt variability vertically and anteroposteriorly^70^.  **Neural control -** Changes might be interpreted as compensatory adjustment to facilitate balance control^71^.  **Modulation during dual task -** Greater on the DT than on the ST.  **DTE -** Higher or positive DTE suggests worse performance on the DT than on the ST. |
| **Rhythm domain** |
| **Stride time (s).**  **Definition -** Length of time it takes to complete one full gait cycle – from one heel strike to the next heel strike with the same foot.  **Marker -** Important metric in assessing walking performance, also in relation to neurological condition^58^.  **Neural control -** A positive correlation exists between stride-time and activation in the primary motor cortex^59^ connected to the sensorimotor area^60^.  **Modulation during dual task -** Greater on the DT than the ST.  **DTE -** Higher or positive DTE indicates worse performance on the DT than on the ST.  **Swing time (s).**  **Definition -** Duration of gait cycle when foot is not in contact with the ground (toe-off) and is swinging forward to prepare for the next step (just before heel strike).  **Marker -** Marker of dynamic balance and gait performance ^62,63^.  **Neural control -** Involvement of brain regions and neural circuits that control leg movements during the swinging phase of walking. Key components: motor cortex for planning and executing movements, basal ganglia for coordination, cerebellum for motor precision, spinal cord circuits for rhythmic muscle activity, and sensory feedback for environmental awareness^62^.  **Modulation during dual task -** Slower on the DT than on the ST.  **DTE -** Higher or positive DTE indicates better performance on the DT than on the ST.  **Double support (s).**  **Definition -** Portion of gait cycle during which both feet are in contact with the ground simultaneously.  **Marker -** Marker of conservative gait strategy for neurological patients compared with healthy subjects.  **Neural control -** Related to damaged locomotor synergies, gait unsteadiness and fall risk^65,67^; longer double support time associated with reduced gray matter volume in dorsolateral prefrontal cortex, right parietal lobules, right motor cortex and sensorimotor cortex^68^.  **Modulation during dual task -** Cognitive DT increases this parameter^69^.  **DTE -** Higher or positive DTE indicates worse performance on the DT than on the ST. |
| **Phase domain** |
| **Swing time (%)**  **Definition -** Percentage of gait cycle when the foot is not in contact with the ground and is swinging forward to prepare for the next step.  **Marker -** Marker of conservative gait strategy in neurological patients compared with healthy subjects; related to damaged locomotor synergies, gait unsteadiness, and fall risk^64^.  **Neural control -** Involving coordinated activity across several brain regions and neural circuits, including: the motor cortex, responsible for planning and executing movements; the basal ganglia, crucial for coordination; the cerebellum, which ensures motor precision; spinal cord circuits, that facilitate rhythmic muscle activity; and mechanisms for sensory feedback, crucial for environmental awareness.  **Modulation during dual task -** Lower percentage on the DT than on the ST; reduced also when balance is poor ^64^.  **DTE -** Higher or positive DTE indicates better performance on the DT than on the ST. |
| **Variability measures** |
| **Swing time variability.**  **Definition -** Coefficient of variation based on the formula: swing time variability = 100 · (standard deviation⁄average swing time)^32^.  **Marker -** Marker of consistency of the swing time among strides. Measure of poor balance and reduced automaticity related to gait unsteadiness and fall risk^65^.  **Neural control -** Determined predominantly by balance-control mechanisms^40^; automatic process in physiological conditions but as an attention-demanding task and executive function^65;^ correlated with grey matter volume^66^.  **Modulation during dual task -** Greater on the DT than on the ST in neurological conditions but not in healthy controls^40^.  **DTE -** Higher or positive DTE indicates worse performance on the DT than on the ST.  **Stride time variability.**  **Definition -** Coefficient of variation according to the formula: stride time variability = 100 · (standard deviation⁄average stride time)^32^.  **Marker -** Marker of rhythmicity and automaticity related to gait unsteadiness and fall risk^61^.  **Neural control -** Under physiological conditions, an automatic process determined predominantly by gait- patterning mechanisms (repeated sequential contraction and relaxation of muscle groups resulting in walking^40^; attention-demanding task involving executive function.  **Modulation during dual task -** Greater on the DT than on the ST^40^ in neurological conditions but not in healthy controls.  **DTE -** Higher or positive DTE indicates worse performance on the DT than on the ST. |

Legend: DTE, dual-task effect; DT, dual task; ST, single task.

**Supplementary Table 2.** Area under the curve (AUC) for each gait measure discriminating between FGDs patients and healthy controls stratified by sex (Single task; Dual task: motor mDT, cognitive cDT, visual-fixation vDT; Dual-task Effect %: motor mDTE, cognitive cDTE, visual-fixation vDTE).

| **Gait measure** | **Sex** | **ST** | **mDT** | **cDT** | **vDT** | **mDTE** | **cDTE** | **vDTE** |
| --- | --- | --- | --- | --- | --- | --- | --- | --- |
| **Pace domain** |  |  |  |  |  |  |  |  |
| Gait speed (cm/s) | Male  Female | 0.941  0.955 | 0.935  0.969 | 0.898  0.945 | 0.947  0.959 | 0.653  0.629 | 0.703  0.672 | 0.607  0.447 |
| Stride length (cm) | Male  Female | 0.935  0.917 | 0.867  0.943 | 0.919  0.933 | 0.926  0.930 | 0.607  0.646 | 0.567  0.614 | 0.464  0.568 |
| Step duration (s) | Male  Female | 0.718  0.916 | 0.816  0.942 | 0.786  0.896 | 0.799  0.927 | 0.619  0.568 | 0.622  0.667 | 0.610  0.542 |
| **Rhythm domain** |  |  |  |  |  |  |  |  |
| Stride time (s) | Male  Female | 0.715  0.913 | 0.817  0.944 | 0.793  0.894 | 0.808  0.927 | 0.625  0.575 | 0.641  0.671 | 0.616  0.548 |
| Swing time (s) | Male  Female | 0.480  0.646 | 0.588  0.755 | 0.638  0.674 | 0.573  0.699 | 0.625  0.607 | 0.594  0.590 | 0.604  0.451 |
| Double support (s) | Male  Females | 0.861  0.973 | 0.907  0.964 | 0.919  0.947 | 0.924  0.966 | 0.697  0.498 | 0.737  0.575 | 0.662  0.530 |
| **Phase domain** |  |  |  |  |  |  |  |  |
| Swing time % | Male  Female | 0.827  0.954 | 0.856  0.923 | 0.884  0.936 | 0.892  0.953 | 0.415  0.444 | 0.693  0.623 | 0.604  0.475 |
| **Variability measures** |  |  |  |  |  |  |  |  |
| Swing time SD | Male  Female | 0.755  0.906 | 0.794  0.909 | 0.771  0.832 | 0.831  0.852 | 0.582  0.561 | 0.662  0.528 | 0.656  0.508 |
| Swing time variability | Male  Female | 0.768  0.896 | 0.758  0.900 | 0.765  0.827 | 0.817  0.844 | 0.582  0.558 | 0.635  0.538 | 0.666  0.496 |
| Step duration SD | Male  Female | 0.735  0.899 | 0.723  0.897 | 0.656  0.833 | 0.757  0.852 | 0.539  0.500 | 0.455  0.454 | 0.574  0.522 |
| Double support SD | Male  Female | 0.718  0.885 | 0.762  0.856 | 0.746  0.799 | 0.763  0.824 | 0.567  0.508 | 0.582  0.527 | 0.582  0.501 |
| Stride time SD | Male  Female | 0.721  0.856 | 0.758  0.854 | 0.662  0.821 | 0.811  0.827 | 0.551  0.542 | 0.466  0.549 | 0.697  0.498 |
| Stride time variability | Male  Female | 0.687  0.814 | 0.700  0.815 | 0.635  0.787 | 0.774  0.794 | 0.529  0.535 | 0.508  0.484 | 0.678  0.511 |
| Stride length SD | Male  Female | 0.662  0.706 | 0.718  0.740 | 0.697  0.660 | 0.715  0.614 | 0.567  0.515 | 0.548  0.549 | 0.526  0.566 |

Legend: s, seconds; cm, centimeters; ST, single task; mDT, motor dual task; cDT, cognitive dual task; vDT, visual-fixation dual task; SD, standard deviation; DTE, dual-task effect.
